# The Beat

**Published:** 2011-03

**Authors:** Erin E. Dooley

## Sunscreen Ingredient Linked to Skin Tumors

An independent science advisory board voted in late January 2011 to confirm a draft National Toxicology Program (NTP) assessment^1^ that concluded retinyl palmitate promotes the development of skin tumors and lesions when applied to the skin in the presence of sunlight. Retinyl palmitate, a form of vitamin A, is used as an anti-aging ingredient in more than 200 sunscreens and other personal care products. In 2010 the Environmental Working Group published an analysis of the NTP’s raw study data that drew the same conclusions.^2^

## Clorox Discloses Ingredients

**Figure:**
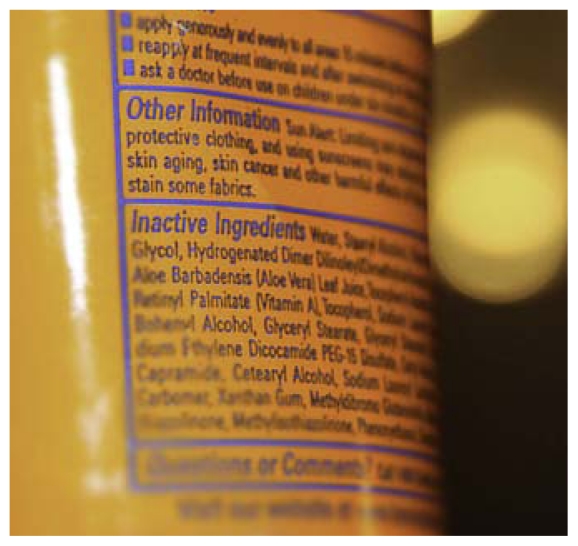
Retinyl palmitate is used in more than 200 products.

In a significant first for a mainstream manufacturer, the Clorox Company has announced it will disclose not only the active ingredients but also specific preservatives and dyes used in each of its cleaning, disinfecting, and laundry products.^3^ The company has also published a roster of fragrance ingredients used in its products (although they are not linked to specific products). Going forward, Clorox product labels will direct consumers to a website where they can find ingredient lists plus Material Safety Data Sheets for formulated products. Manufacturers are not required by the Consumer Product Safety Commission (which regulates cleaning supplies and laundry products) to list either the term “fragrance” or fragrance ingredients on labels or Material Safety Data Sheets.^4^

## Traffic Noise and Stroke Risk in Older Adults

A study of more than 57,000 Danish residents over age 50 has revealed an association between exposure to loud traffic noise and increased risk for stroke.^5^ For every 10-decibel increase in estimated road traffic noise, the relative risk of stroke rose 14% overall and 27% among people over age 64.5 after adjusting for multiple stroke risk factors. Sleep disturbances can contribute to cerebro- and cardiovascular risks, and elderly people are already more susceptible to broken sleep, which could help explain why the association was strongest among the oldest participants.

## California Mercury Sediments: Let Sleeping Dogs Lie . . . for Now

Some California waterways are contaminated with mercury-laden sediment, a remnant of the state’s gold mining legacy. Although the mercury-contaminated sediment poses little threat if it remains buried, disturbed sediment can release inorganic mercury that can convert to toxic methylmercury. A feasibility study predicts that removing such sediment with current suction-dredging technology would exacerbate the mobilization of mercury—fine-grained sediment particles are the sediment fraction with the greatest mercury concentration as well as the most likely both to elude standard recovery equipment and to travel far downstream.^6^

## Updated Guidance on CFL Cleanup

**Figure:**
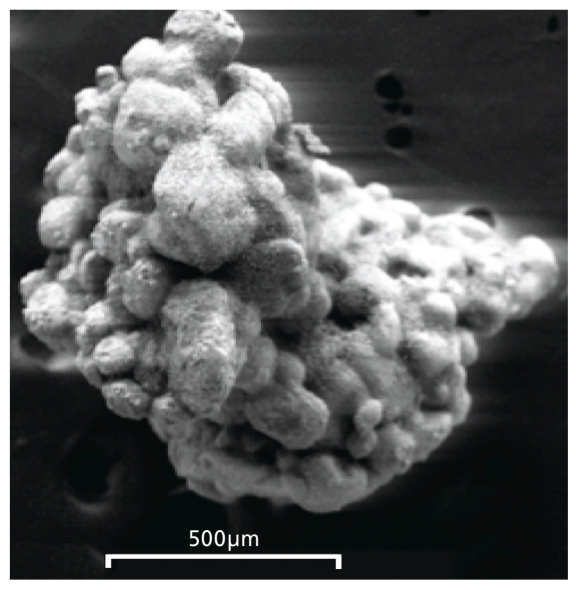
Grains of gold and mercury amalgam

The U.S. EPA recently released updated its guidance for consumers on preventing breakage of mercury-bearing compact fluorescent lamps (CFLs) as well as storage, handling, usage, and cleanup of hard surfaces and carpets.^7^ Among other revisions, the EPA now advises consumers to wait 5–10 minutes after a bulb breaks before cleaning it up, rather than the 15 minutes recommended in earlier agency guidelines. This change is based on a 2008 study showing that most of the mercury in broken CFLs was released within 5 minutes.^8^
